# Parasitofauna and current status of anthelmintic resistance in Latvian sheep farms

**DOI:** 10.14202/vetworld.2022.414-418

**Published:** 2022-02-23

**Authors:** Dace Keidāne, Alīna Kļaviņa, Marta Barbara Bergmane, Līga Kovaļčuka

**Affiliations:** 1 Institute of Food and Environmental Hygiene, Faculty of Veterinary Medicine, Latvia University of Life Sciences and Technologies, K. Helmana street 8, Jelgava, LV-3004, Latvia; 2 Clinical Institute, Faculty of Veterinary Medicine, Latvia University of Life Sciences and Technologies, K. Helmana street 8, Jelgava, LV-3004, Latvia

**Keywords:** anthelmintic resistance, gastrointestinal parasites, sheep

## Abstract

**Background and Aim:**

Parasitic invasions, especially gastrointestinal nematodes, are widespread and are one of the main problems in sheep farms. For this reason, sheep are dewormed more often than other livestock species, often several times a year. Concerns about antiparasitic resistance from the farmers and veterinarians have arisen because, on some farms, antiparasitic drugs are used routinely for very long periods. There are no data available on anthelmintic resistance in gastrointestinal nematodes in sheep in Latvia. Our work aimed to determine the most common endoparasites in sheep and the degree of anthelmintic resistance on sheep farms in Latvia.

**Materials and Methods:**

All sheep (577) underwent a coprological examination before the start of the study, and only sheep diagnosed with more than 200 McMaster eggs per gram of feces were included in the study. A fecal egg count reduction (FECR) test was performed on 20 sheep flocks in Latvia.

**Results:**

In Latvia, sheep were most commonly infected with *Eimeria* spp. 97% (confidence interval [CI] 95% 96-98). The second most commonly diagnosed species were *Trichostrongylidae* 91% (CI 95% 89-93) and *Strongyloides* spp. 76% (CI 95%, 72-79). The ivermectin (IVM) FECR was 0.74% (0.73-0.74), showing resistance in all sheep farms included in this study. Albendazole (ABZ) FECR 0.89% (0.88-0.89) was effective.

**Conclusion:**

This study showed that the most popular deworming drugs (ivermectin, albendazole) in Latvia are ineffective in sheep. Additional studies on the use of IVM+ABZ combinations for deworming sheep should be performed.

## Introduction

Sheep are often infected with Trichostrongylidae (*Haemonchus* spp., *Ostertagia* spp., and *Trichostrongylus* spp.) [1,2]. Among the cestodes, the current invasion of sheep is monieziosis. Protozoa dis­ease, eimerosis is more common in Latvia in lambs. Latvia is not an exception among European countries and they are also troubled by the same types of parasites. Due to climate change, sheep farms are increasingly facing problems caused by these parasites. The sheep are often clinically ill with diarrhea, reduced body weight, developmental delays, and death [1].

Parasitic invasions and their possible control are widespread concerns in veterinary medicine, especially on sheep farms. Studies in many countries have shown that anthelmintic resistance in sheep farming is a global problem. The first reports of anthelmintic resistance appeared in the early 1990s [3]. The current research has also been conducted in European countries such as Austria, Denmark, Italy, Spain, the Netherlands, the Czech Republic, Greece, and Lithuania [4-9]. Anthelmintic resistance seems to be quite common in practical veterinary medicine. However, unfortunately, there have been no studies conducted in Latvia thus far. It should be noted that there are few deworming products for sheep registered in Latvia; ivermectin (IVM), levamisole (LEV), albendazole (ABZ), closantel, and monepantel are available for deworming in sheep. IVM (macrocyclic lactone) and ABZ (benzimidazole) are more commonly used [10]. IVM is also used to control ectoparasites, but ABZ is used to control monieziosis.

No less relevant is the issue of methods for detecting antiparasitic resistance. The currently used method for antiparasitic detection is counting parasitic eggs before and after deworming – the fecal egg count reduction test (FECRT). This method is used by most scientists [4,6,9,11,12]. Recently, publications have appeared where FECRT is combined with molecular testing methods. Some authors also suggest combining molecular methods with FECRT [8,13,14].

This study aimed to determine the most common endoparasites in sheep and the degree of anthelmintic resistance in sheep farms in Latvia.

## Materials and Methods

### Ethical approval and Informed consents

Ethical approval was not required to conduct this study. In all cases, written informed consent was obtained from the farm owners for the study.

### Study period, location, and the sampling procedure

This study was conducted in September and October 2019 and 2020. In this study, 20 sheep farms from all regions of Latvia were included (six in Vidzeme, eight in Kurzeme, three in Zemgale, and three in Latgale). The sheep farms were divided into small farms, where the number of sheep was less than or equal to 100 animals (n=7), average with 100-300 animals (n=10), and large farms with more than 300 sheep (n=3). On each farm, depending on the size of the farm, study groups of sheep were created (ewes that were 3 or more years old). On small farms, 10% of the sheep from the total herd were included in this study, while it was up to 20% from medium and large farms. All animals underwent a coprological examination before the start of the study, and to obtain statistically relevant data, only sheep diagnosed with more than 200 McMaster eggs per gram of feces (EPGs) were included in the study [13].

To evaluate the effectiveness of deworming agents, a coprological sample was taken rectally from each sheep before deworming and 14 days after deworming. Coprological samples were taken while wearing polyethylene gloves, labeled, and placed in cold boxes. The samples were delivered to the laboratory within a day and examined within 2 days. In the laboratory, the coprological samples were stored in a refrigerator at 4°C until the time of analysis.

### Treatment groups and medications

For sheep deworming, registered anthelmintic medications were used chosen by the farmer or the veterinarian. If the farm had been using a particular drug for several years, we used the same drug on the sheep on each particular farm.

In this study, 10 farms used IVM 1% 0.2 mg/kg (Biomectin 1%, Vetoquinol Biowet, Poland), 10 farms used ABZ 10% 5 mg/kg (Albex 10%, Chanelle, UK), and three farms used an IVM 1% 0.2 mg/kg/ABZ 10% 5 mg/kg combination. Initially, the study included farms that used Ivomec Plus (IVM 1% and clorsulon 10%), closantel 50 mg/ml, monepantel 25 mg/ml, and LEV 11.8% for deworming sheep. Due to the insufficient number of animals in each group, these farms were not included in the analyses.

### Statistical analysis

To determine the parasitofauna on the farms, we examined the coprological samples of sheep according to the methods ofMcMaster and Baermann [13]. For the diagnosed parasites, we determined the invasion prevalence in percentages and the invasion intensity (total egg count per sheep) in the averages. Coprological samples of sheep taken both before and on the 14^th^ day were examined by the McMaster method. We took 4 g of sheep feces mixed with 56 ml NaCl (density 1.20 [0.5 moL/L]). We examined the McMaster chamber and calculated the worm load according to the standard formula. Individual FECs, expressed in EPG, were calculated using the McMaster technique. The mean FECR for each group and the corresponding 95% confidence intervals (CIs) were calculated as follows:

FECR%=100%×(1-m^2^/m1),

whereas, m1=arithmetic mean FEC across the animals excreting eggs before treatment and m2=arithmetic mean FEC after treatment [5,15].

Three result labels were developed to interpret the results based on Coles *et al*. [12]. If the FECR <95%, then the farm is dominated by a population of resistant (R) Trichostrongylidae. In contrast, if the FECR is ≥95%, then Trichostrongylidae is susceptible (S). Results that do not fall within these ranges are referred to as the suspended population (R/S).

## Results

This study showed that sheep in Latvia are infested with parasites such as *Eimeria* spp., Trichostrongylidae, *Strongyloides* spp., *Trichuris* spp., *Capillaria* spp., *Moniezia* spp., and Protostrongylidae. The prevalence % of the diagnosed parasites is shown inTable-1.

**Table 1:** Invasion prevalence % and invasion intensity in sheep farms in Latvia.

Invasion	Prevalence%, (95% CI)	Invasion intensity (95% CI)	Positive sheep/total number of sheep	Eggs count (min-max)
*Eimeria* spp.	97% (96-98)	1478 (190 – 2766)	290/577	(50-178950)
Trichostrongylidae	91% (89 – 93)	1056 (821 – 1290)	331/577	(50-23750)
*Strongyloides* spp.	76% (72 – 79)	215 (111 – 319)	59/577	(50-2600)
*Trichuris* spp.	0,5% (0,1 – 1,5)	63 (23 – 102)	4/577	(50-100)
*Capillaria* spp.	0,2% (0 – 1)	75 (0 – 393)	2/577	(50-100)
*Moniezia* spp.	77% (73 – 80)	616 (199 – 1033)	44/577	(50-7550)
Protostrongylidae	0,2% (0 – 1)	2 (0 – 79)	2/577	(1-2)

Sheep were most commonly infected with *Eimeria* spp. 97% (CI 95% 96-98). The second most commonly diagnosed infestations were Trichostrongylidae 91% (CI 95% 89-93) and S*trongyloides* spp. 76% (CI 95% 72-79). On some farms, Trichostrongylidae invasions recur yearly and cause significant economic losses to the animal owners. The third most common infestation in sheep farms was *Moniezia* spp. 77%. *Moniezia* spp. invasion on sheep farms is often diagnosed, and although clinical signs are rare in adult sheep, they cause serious health problems in lambs, which are clinically manifested by diarrhea, weight loss, developmental delays, and even animal death. Less frequently, we diagnosed *Trichuris* spp. invasion 0.5%, C*apillaria* spp. 0.2%, and Protostrongylidae 0.2%. The intensity of the invasion is shown in Table-1.

The highest intensity of invasion was in sheep invaded with *Eimeria* spp. in the average of 1478 (95%CI 190-2766). Eimeria was diagnosed on all study farms (p=0.0792). The intensity of Trichostrongylidae invasion was an average of 1056 (95%CI 821-1290), and this invasion, similar to *Eimeria*, was diagnosed on all sheep farms included in this study (p=0.0674). The intensity of *Trichuris* invasion was an average of 63 (95% CI 23-102), and this invasion was diagnosed on two farms. On one farm, *Trichuris* invasion was diagnosed in one sheep, and on another farm, three sheep were diagnosed. We diagnosed *Moniezia* invasion from cestodes with an average of 616 (95%CI 199-1033). During the study, only five farms were not diagnosed with monieziosis in the coprological samples taken before or after deworming (p=1.2800). In one farm, in two animals, *Capillaria* with an average invasion intensity of 75 (95% CI 0-393) were diagnosed (according to McMaster). Protostrongylidae was diagnosed in two sheep farms, incomplete four sheep, and the average invasion intensity was 2 (95%CI 0-79). The most common invasions that cause economic losses in sheep farms in Latvia are nematodes of the family Trichostrongylidae. The effectiveness of the deworming agents on sheep farms is shown in Table-2.

**Table 2:** Anthelmintic efficacy of sheep farms.

Sheep farms	Anthelmintic class/ No. of animals included	Mean EPG value before/after treatment	FECR% (95% CI)	Status
1	IVM/18	786/106	0.87 (0.81 – 0.90)	R
2	IVM/9	361/78	0.78 (0.63 – 0.88)	R
3	IVM/20	1020/187	0.81 (0.77 – 0.86)	R
4	IVM/44	1577/515	0.68 (0.64 – 0.71)	R
5	IVM/15	290/13	0.95 (0.89 – 0.99)	SR
6	IVM/8	1888/806	0.56 (0.54 – 0.60)	R
#	ABZ/20	1887/312	0.83 (0.82 – 0.84)	R
7	IVM/15	477/40	0.92 (0.85 – 0.95)	SR
8	IVM/18	644/279	0.56 (0.53 – 0.60)	R
#	ABZ/15	1140/103	0.91 (0.87 – 0.94)	SR
#	IVM+ABZ/20	903/100	0.89 (0.85 – 0.92)	R
9	IVM/20	3370/905	0.73 (0.68 – 0.77)	R
10	IVM/18	3231/678	0.79 (0.76 – 0.82)	R
11	ABZ/20	460/130	0.72 (0.62 – 0.80)	R
12	ABZ/18	672/39	0.94 (0.90 – 0.97)	S
13	ABZ/16	938/119	0.87 (0.86 – 0.89)	R
14	ABZ/20	945/10	0.99 (0.98 – 100)	S
15	ABZ/15	530/0	100 (0.98 – 100)	S
16	ABZ/20	1562/247	0.84 (0.81 – 0.87)	R
17	ABZ/20	767/80	0.90 (0.88 – 0.91)	SR
18	ABZ/20	613/3	100 (0.98 – 100)	S
19	IVM+ABZ/20	825/32	0.96 (0.93 – 0.98)	S
20	IVM+ABZ/20	637/0	100 (100 – 100)	S

IVM-Ivermectin; ABZ – Albendazol; IVM + ABZ combination; Mean eggs per gram of faeces (EPG); faecal eggs count reduction percentages (FECR%) and 95% confidence intervals (CI); status R – resistance, S –susceptible, SR – suspected of resistance

For sheep deworming, we used 0.2 mg/kg IVM subcutaneously, 5 mg/kg ABZ orally, and an IVM+ABZ combination. Among the 10 sheep farms included in the study that used IVM, two farms did not show anthelmintic resistance when calculating FECR%. However, when calculating CI, farm No. 5 showed 0.95% (0.89-0.99%) and farm No.7 0.92% (0.85-0.95%), which means that there may be resistance in a larger number of animals. As a result, the parasites may have developed resistance on all sheep farms where IVM was used. Ten farms that used ABZ for deworming sheep were tested for resistance; four farms were free of resistance, and resistance was questionable in two (farm No. 17 FECR% 0.90 [0.88-0.91] and farm No.8 0.91 [0.87-0.94]). In three farms, where sheep were dewormed with IVM+ABZ combinations, one farm showed FECR resistance of 0.89% (0.85-0.92%), but farms No. 19 and No. 20 had no resistant parasites. Farm No. 8, which used IVM, ABZ, and IVM+ABZ combinations, had resistance against all drugs included in this study. In addition, farm No. 6, IVM and ABZ were ineffective. It should be noted that farms with resistance to the above drugs had been using them consistently for 5 years or more. It is also important to note that deworming was carried out several times a year on farms with resistance. On farms, where no resistance was found, deworming was performed only once or twice a year (in spring and autumn), and sheep breeding was carried out for a relatively short time.

## Discussion

This study aimed to determine the most common endoparasites in sheep and the degree of anthelmintic resistance in sheep farms in Latvia. The data obtained in this study on the most common sheep parasitosis are to some extent similar to those from other countries [2,6,16-18]. In Latvia, *Eimeria* spp. 97% and Trichostrongylidae invasion are quite common in sheep, *Strongyloides* spp. is less often diagnosed at 76% and *Moniezia* spp. at 77% are diagnosed less frequently. In Sweden, the invasion intensity of some sheep flocks in *Haemonchus* was 90-100%; however, *Eimeria* was diagnosed in all sheep flocks studied [19], Rinaldi *et al*. [20] noted that in Austria, the extent of *Haemonchus* invasion in sheep farms was 77%; in Italy, it was 73%; and in Ireland, it was only 4%. In Spain, studies on sheep have shown an intensity of invasion of up to 100% for nematodes of the family Trichostrongylidae [2]. So far, there are no data on which species of Trichostrongylidae (*Haemonchus, Ostertagia*, or *Trichostrongylus*) predominate in sheep farms in Latvia. Obviously, as the climate changes animal movement between countries increases, these invasions will have to be increasingly considered in the future. The lack of availability of effective anthelmintics is a cause for concern.

This study on antiparasitic resistance in sheep was conducted for the 1^st^ time in Latvia. We found that the most commonly used drugs in Latvia for the control of Trichostrongylidae are IVM and ABZ. Less commonly used are monepantel, LEV, and closantel. In our study, these drugs were used on only three sheep farms. Trichostrongylidae invasions are resistant to IVM and ABZ worldwide, including in Latvia, potential alternative medicines options and prevention measures in sheep farms is becoming prominent. Research on antiparasitic resistance is emerging worldwide [9,21-26]. Major sheep farms in Europe have noted anthelmintic resistance. As mentioned, resistance to ABZ in sheep is particularly notable in many European countries [1,9,21,23,25,26], where resistance to ABZ has also been reported. In addition to ABZ resistance, there are studies of LEV and IVM resistance in the UK [1,27,28]. Our study found antiparasitic resistance on essentially all 10 farms that regularly used IVM. Sheep farms using ABZ performed better. This is probably because IVM is used more often in Latvia for deworming sheep than ABZ, historically, and as the price difference between the two drugs is also significant. The FECR was above 90% in five farms, while the FECR was below 90% in five other farms. Among the three farms included in this study that used the IVM+ABZ combination as an anthelmintic, we found FECRs lower than 90% in one farm. In Lithuania, IVM resistance was detected in two of 16 sheep farms, and FBZ resistance was detected in three farms [29]. The situation is similar in similar studies in Belgium [4], the Netherlands [5], Denmark [6], Austria [30], France, Greece, and Italy [31].

In parallel with this study, another study was performed to investigate parasite prevention measures in sheep farms. Preliminary data from this study show that the most common errors during deworming sheep are excessive use, repeatedly using antihelmintic drugs at the inappropriate dose, administration route, ignoring official storage, and use rules [32].

## Conclusion

This study showed that the most popular deworming drugs in Latvia are ineffective in sheep. The IVM FECR was 0.74% (0.73-0.74), showing resistance in all sheep farms included in this study. ABZ FECR 0.89% (0.88-0.89) was effective. Additional studies on the use of IVM+ABZ combinations for deworming sheep should be performed. The limitation of the study, is that we used a small number of farms. The authors suggest a future study with a more number of farms involvement for the in-depth information.

## Authors’ Contributions

DK and LK: Conceptualization, methodology, and writing of the original draft. LK, DK, AK, and MBB: Sample collection, laboratory work, data curation, equally conceived the work with literature, and drafted and reviewed the manuscript. LK, DK, and AK: Drafted and revised the manuscript. All authors have read and approved the final manuscript.
